# Ethnopharmacology, Biological Evaluation, and Chemical Composition of *Ziziphus spina*-*christi* (L.) Desf.: A Review

**DOI:** 10.1155/2022/4495688

**Published:** 2022-05-29

**Authors:** Mahmoud Dogara Abdulrahman, Ali Muhammad Zakariya, Harmand A. Hama, Saber W. Hamad, Sawsan S. Al-Rawi, Sarwan W. Bradosty, Ahmad H. Ibrahim

**Affiliations:** ^1^Department of Biology, Faculty of Education, Tishk International University, Erbil, Kurdistan Region, Iraq; ^2^Institute of Biological Sciences, University Malaya, Kuala Lumpur, Malaysia; ^3^Department of Biological Sciences, Sule Lamido University Kafin Hausa, Jigawa State, Nigeria; ^4^Department of Field Crops, College of Agricultural Engineering Sciences, Salahaddin University, Erbil, Kurdistan Region, Iraq; ^5^Department of Community Health, College of Health Technology, Cihan University, Erbil, Kurdistan Region, Iraq; ^6^Pharmacy Department, Faculty of Pharmacy, Tishk International University, Erbil, Kurdistan Region, Iraq

## Abstract

Medicinal plants are the primary raw materials used in the production of medicinal products all over the world. As a result, more study on plants with therapeutic potential is required. The tropical tree *Ziziphus spina* belongs to the Rhamnaceae family. Biological reports and traditional applications including management of diabetes and treatment of malaria, digestive issues, typhoid, liver complaints, weakness, skin infections, urinary disorders, obesity, diarrhoea, and sleeplessness have all been treated with different parts of *Z. spina* all over the globe. The plant is identified as a rich source of diverse chemical compounds. This study is a comprehensive yet detailed review of *Z. spina* based on major findings from around the world regarding ethnopharmacology, biological evaluation, and chemical composition. Scopus, Web of Science, BioMed Central, ScienceDirect, PubMed, Springer Link, and Google Scholar were searched to find published articles. From the 186 research articles reviewed, we revealed the leaf extract to be significant against free radicals, microbes, parasites, inflammation-related cases, obesity, and cancer. Chemically, polyphenols/flavonoids were the most reported compounds with a composition of 66 compounds out of the total 193 compounds reported from different parts of the plant. However, the safety and efficacy of *Z. spina* have not been wholly assessed in humans, and further well-designed clinical trials are needed to corroborate preclinical findings. The mechanism of action of the leaf extract should be examined. The standard dose and safety of the leaf should be established.

## 1. Introduction

People have resorted to natural sources for cures for various ailments since ancient times [1, 2]. For millennia, people throughout the world have relied on medicinal herbs or plants [3]. Even more impressively, almost 25% of modern drugs are derived from the stem of plants in some way. This shows a robust foundation for plant-derived medicines [4]. Many current medications, nutraceuticals, nutritional supplements, and pharmaceutical products are based on these compounds. Since the development of bacterial and fungal resistance and many other diseases has become an increasing concern, there has been an upsurge in interest in the therapeutic capabilities of traditional medicines. No extensive reviews of *Z. spina* have been found, according to our literature search. There is only one attempt to review the plants in 2012 [5]. Ethnopharmacology, origin and distribution, taxonomic, morphological, biological evaluation, and chemical composition are examined in this study, which provides a complete overview and up-to-date information on the therapeutic properties of *Z. spina* with emphasis on its biological activity and chemical composition.

## 2. Materials and Methods

### 2.1. Search Criteria

#### 2.1.1. Inclusion criteria

Electronic databases such as ScienceDirect, PubMed, Wiley, Google Scholar, Hindawi, and Springer extracting valuable information from original scientific research papers were used to find articles on *Z. spina*. These and many more biological evaluations were utilized as key phrases in this research. These included “antifungal and antibacterial”, “anti-inflammatory”, “herbal”, and “anticancer”.

#### 2.1.2. Exclusion criteria

**Figure 1:**
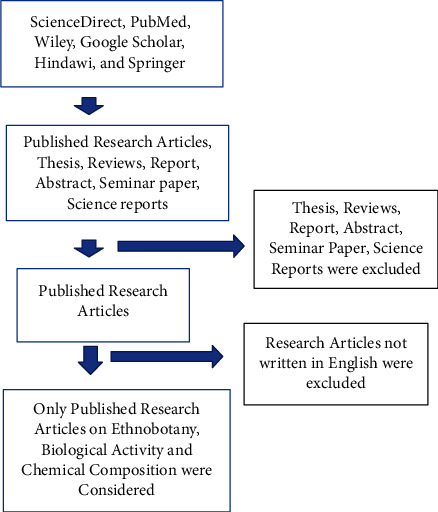
Flowchart of the methodology.

Data from questionable online sources, as well as thesis reports and review publications, were excluded from this investigation (Figure 1).

## 3. Results and Discussion

### 3.1. Ethnopharmacology

**Figure 2:**
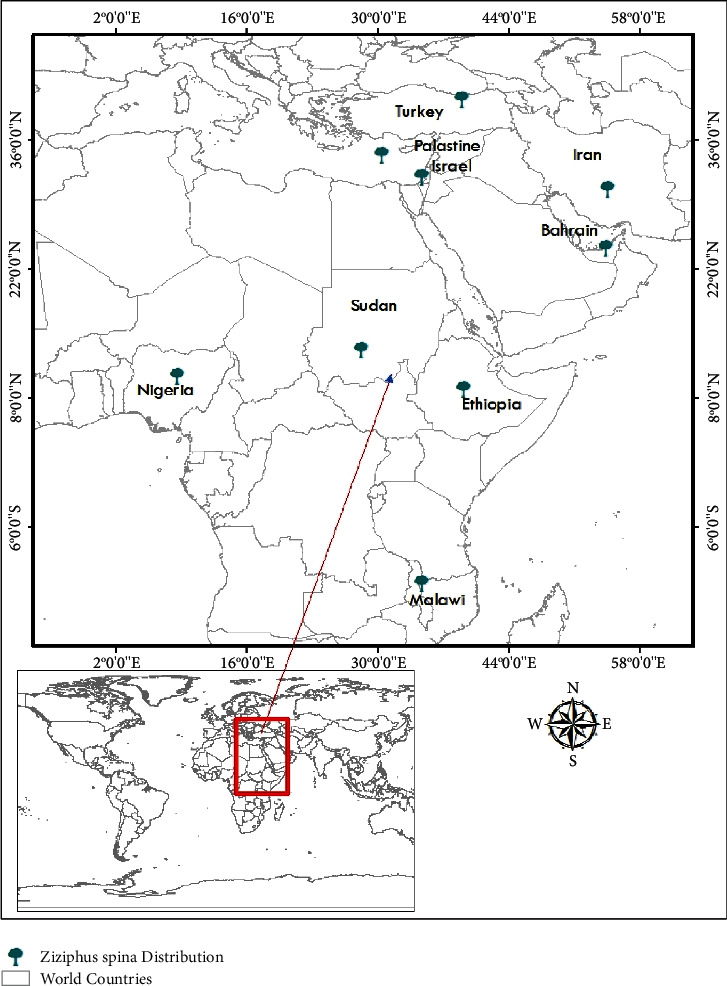
Distribution of *Ziziphus spina*.

For the majority of human history, people have relied on local flora to heal a broad range of maladies, both those of themselves and their domesticated animals [6]. Depending on the community's culture and religious beliefs, some of the plants were also used for religious rituals [3]. Traditional medicine as a collection of practices and knowledge gathered from the observations and practical experiences of previous generations was described and used for the diagnosis, eradication, and prevention of physical and nonphysical sickness [7]. *Ziziphus spina* has been traditionally reported in different parts of the world (Figure 2) for the treatment of various ailments [8, 9]. Flowers, leaves, and roots were reported in traditionally treated stomach pain, a disorder in Malawi, Iran, and Sudan [9–11]. In traditional medicine and as a source of nourishment and energy, this species is well known [12]. The extract of the plant is used in the management of dandruff, wounds, and hair loss in Bahrain [13]. In Palestine, the leaves are used in the treatment of skin infections [14]. As a remedy for constipation, people in Turkey rely on the fruit's fiber content [15]. Cough medicine in Nigeria is typically made from the roots [16]. Fruits are used in Sudan to treat diarrhoea, rheumatism, scorpion stings, malaria, and antispasmodics [8]. Decoction is made by boiling leaves and fruits in water for half an hour, and then it should be taken three times a day as an oral supplement to lower cholesterol and cancer risk. Boiling leaves and fruits in water for half an hour produces a typical decoction that is taken three times per day as an oral supplement [12]. All parts of the plants are traditionally used in the treatment and management of various ailments in different parts of the world [9–11].

#### 3.1.1. Origin, Taxonomic, and Morphological Description

In southern Sudan, Ethiopia, Northern Lebanon, and Syria, the perennial *Z. spina*, often known as Christ's thorn, grows [12, 17]. A number of *Ziziphus* species are naturally suited to dry and hot temperatures, which makes them appropriate for cultivation in tough situations with degraded soil and inadequate water supply [17]. It is a Sudanese-bred tropical evergreen tree. It may be found in every valley and plain in Israel and is generally found at low altitudes [18]. It is a spiky and hardy, tiny shrub or tree with thorns that can withstand heat and dehydration [19]. Although this plant normally matures into a tree, heavy grazing in the latter dry seasons sometimes results in it becoming a shrub instead [19]. More than 170 species of shrubs and small trees are found in warm temperate and subtropical locations of the globe [20]; for example, Ziziphus spina-christi (L.) Desf., synonymous Ziziphus spina*-*christi var. *spina -c hristi*, *Rhamnus spina -c hristi* L., and infraspecific taxa of Ziziphus spina-*c*hristi var. *aucheri* (Boiss.) Qaiser and Nazim. Up to a 45 cm trunk diameter is possible for this tree, which may grow to a height of 5–10 meters [19]. The bark is extensively fissured and yellowish brown or light grey in colour. Round or oval in shape, the crown's thick branches stretch out widely and weep at the ends.

### 3.2. Biological Evaluation

Alternative medicine is based on the use of medicinal plants, which has led to the development of many novel pharmaceuticals [21]. Increasingly more than 80% of medicine was derived from plants in the nineteenth century, and the scientific revolution led to the development of the pharmaceutical business, where the manufactured pharmaceuticals became more prominent [22]. There is a greater usage of medicinal plants in the treatment of ailments since they are regarded as safe and effective pharmaceuticals, as well as having fewer side effects and costing less than other drugs [23]. *Z. spina* was subjected to a number of biological evaluations (Table 1).

#### 3.2.1. Antioxidants

Antioxidant plant-based medicine formulations are used to prevent and cure complicated illnesses such as atherosclerosis, stroke, diabetes, Alzheimer's disease, and other neurological disorders [150]. In the human body and food system, free radical reactions occur. In the form of reactive oxygen and nitrogen species, free radicals are a natural element of physiology. The hunt for antioxidants from natural sources has got a lot of attention, and researchers are working hard to find chemicals that may replace synthetic antioxidants [151]. The potential capability of *Z. spina* was evaluated using animal models, DPPH and *β*-carotene-linoleic acid, FRAP, ribosomal degradation test, SRSA, TRPA, ABTS, Rancimat, and many more procedures (Table 1). Antioxidant potentials were found in all assessment techniques (Table 1). The activity revealed high antioxidant ability in terms of radical scavenging activity, with IC_50_ values of 21.4, 24.2, and 54.3 g/mL for methanolic, aqueous, and ethanolic extracts, respectively. The reducing power of the extracts was revealed to be concentration dependant [27]. The plant displayed antioxidant activity with IC_50_ values of 5.5 and 4.1 g/mL [25]. The extracts demonstrated immunologic and antioxidant effects on rabbits subjected to a 2 percent H_2_O_2_ solution to induce oxidative stress, according to our results [32]. The IC_50_ value for scavenging activity was determined to be 53 [36]. The plant extract's naturally occurring antioxidants may be synthesized into nutraceutical that can help prevent oxidative damage in the body.

#### 3.2.2. Anti-Inflammatory

Physical trauma, noxious chemicals, and microbial infections may all produce inflammation, which is the body's natural reaction to protecting itself from further damage. A host of infections, irritants, and damaged tissues are dealt with during this procedure [1, 6]. Many medications are available to combat inflammation, but long-term usage may result in side effects such as nausea, vomiting, bone marrow depression, and fluid or salt retention [6]. A new supply of structurally essential compounds from plants has been discovered by traditional medicine, which means that it is always expanding its horizons [3]. It is well known that plants are rich in chemical compounds. Compositional diversity in plants has gone largely unexploited, and novel lead chemicals for the treatment and management of inflammation might be found. The crude extract was tested in a variety of solvents and shown to be efficient in treating a variety of inflammation-related diseases, as indicated in Table 1, with a total inhibition of 79.2%. This explains why these species have traditionally been used as polyherbs to treat ulcers [46]. High activity was recorded with the methanolic extract even at 95 compared with the standard at 20.2%, respectively [50]. There is a dose-related impact in all models except for tail-flick, which has no statistically significant activity [52]. In terms of histological changes, the group treated with leaf extract had the highest improvement (ointment), whereas the other group, which had early re-epithelialization, had a greater cellular response to the inflammatory process. Burn wounds are frequent in both rich and developing countries; however, in poor countries, burns represent a serious public health issue due to the high frequency of severe sequelae. Burn wound healing is a complicated process that requires little assistance but still produces discomfort, and the wounds are susceptible to infection and other consequences [152]. Burn healing efficiency of *Z. spina* extract was assessed in the rat model (Table 1). To sum it up, ointment from the plant leaf was found to have good promise for speeding up the healing of burn wounds (Table 1). *In vitro* and *in vivo* studies show that *Z. spina* may be used to treat and control inflammation.

#### 3.2.3. Antibacterial

Because of their unique qualities, plant extracts have recently got a lot of interest in terms of producing antibacterial agents. Because there is a rising interest in environmental protection, alternative synthesis processes that are ecologically benign and do not require harmful ingredients are needed. Due to activities such as inappropriate and careless administration of medicines in the clinic, bacterial strains have evolved resistant to a broad spectrum of antibiotics, resulting in the creation of multidrug-resistant microorganisms [153]. The preliminary examination of *Z. spina* against several Gram-positive and Gram-negative bacteria revealed that it was extremely significant. The extract was shown to exhibit action against some of the tested strains at a concentration of 100 mg/mL, with a minimum MIC of 6.25 g/mL for the methanolic extract. These results provide early evidence that crude extracts may be used to treat bacterial infections [56]. Both crystals had antibacterial activity against the tested strains, with SeONPs having greater antimicrobial activity than ZnONPs, according to the findings [24]. The ethanolic extract had the maximum activity against *S. aureus*, with an activity of 18 mm, while the aqueous extract had the lowest activity against *B. subtilis*, with an activity of 13 mm [70]. All examined strains were inhibited by the aqueous stem bark extracts, with the maximum inhibition against *Klebsiella* spp. and *E. coli* at 20 mm and 20 mm, respectively [76]. Because of the current exploratory findings, *Z. spina* extract might be a valuable source for the identification and development of novel antibacterial active compounds. The plant may be used to make effective antibiotics against bacterial infections.

#### 3.2.4. Antifungal

Plant extracts and natural goods are gaining popularity since they do not pose a health risk or pollute the environment [111]. The lack of viable treatment choices, as well as pathogen cross-resistance to the earlier medications fluconazole and itraconazole, has required the search for novel antifungal agents from a variety of sources, including medicinal plants [110]. The following study found the extract exhibited the growth of fungal strains (Table 1). Several research projects have highlighted the antifungal properties of the species (Table 1). It was sensitive at 600 mg/mL and was not inhibited at low dosages, showing that the extract possesses antifungal activity [95]. At a concentration of 128 mg/mL, there was no evidence of any action against the *Candida* species [82]. At a dosage of 100 mg/mL, it showed considerable action with a 20 mm inhibitory zone. These findings might help in the treatment of fungal infections [102]. At a dosage of 500 mg/mL, both ethanolic and aqueous extracts inhibited *Candida albicans* with inhibition diameters of 32 mm and 18 mm, respectively [94]. As a result, it has been reasonable to conclude that the rise in extract concentration has antifungal action is due to the compounds' synergistic impact, which increases the contact area and extract access to the fungal strain.

#### 3.2.5. Antidiarrhoeal Effects

Diarrhoea can be described as an adult's daily bowel movement that surpasses 200 g and contains between 60 and 95% of water. Diarrhoea caused by an infectious agent is the leading cause of newborn mortality in underdeveloped countries [154]. Children under the age of two have been found to have the greatest mortality rates, with a mortality rate of 20 fatalities per 1000 people [154]. Diarrhoea is responsible for more illnesses and deaths in children than any other disease combined in some regions of the world [155]. The World Health Organization has established a Diarrhoeal Disease Control (DDC) program to address the issues of diarrhoea in poor countries. This program involves investigations of traditional medicinal practices [155]. According to the findings, the extract of *Z. spina* protected rats from castor oil-induced diarrhoea and reduced intraluminal fluid collection and gastrointestinal transit. The LD_50_ values for intraperitoneal and oral administration in mice were 3465 and 1200 mg/kg, respectively (Table 1). The findings revealed that the extract may include physiologically active components that are antidiarrhoeal, which could explain its traditional use for gastrointestinal disorders.

#### 3.2.6. Antiparasitic

A major public health problem is parasitic infections, which can cause morbidity and even death in their victims. The use of chemical drugs to combat parasites is effective, but there are drawbacks, such as drug resistance, drug residues, and undesirable side effects. Alternative remedies need to be studied [156]. The leaf extract is effective against Egyptian species of schistosomes at concentrations of 6, 25, 12.5, 25, 50, 100, and 200 mg/mL [118]. The extract of the leaves dramatically reduced the viability of leishmanial parasites at *p* > 0.001, while inducing NO generation and release, apoptosis, and plasma membrane permeability in macrophage cells without causing injury (Table 1). Methanol and aqueous extracts had IC_50_ values of 60 and 80 g/mL, respectively. The extracts had a devastating effect on the parasites (Table 1). Traditional usage of the leaf extract for antiparasitic ailments may have been based on the discovery of physiologically active components in the leaves.

#### 3.2.7. Antiviral

Since the Stone Age, medicinal plants have played an important role in addressing human health difficulties. They help the human body by acting as restorative, defensive, and supporting agents. Because antiviral medications are frequently ineffective in treating viral infections, there is a growing demand for new antiviral agents that can combat viral resistance. New and better antiviral medicines are needed to combat viral infections. Antiviral medications currently on the market are frequently ineffective in treating viral infections because of the issue of viral resistance [157]. A 50-year-old guy was successfully treated with the leaf extract for rashes (Table 1). When compared with the control group, there was a significant reduction in the growth of the rashes [125]. There is a growing demand for the discovery of novel antiviral substances. Only one research was shown to substantially suppress the development of the virus, according to the study (Table 1). Because of this, the study recommended more research into viral disorders.

#### 3.2.8. Antimalarial

Malaria has long been seen as a public health threat around the globe. More than 3.2 billion individuals are in danger of contacting malaria parasites, according to estimates [3]. The histology investigations of the liver and spleen indicated serious abnormalities. Improved histopathology results were shown in animals that had been treated. Biochemical tests showed a considerable recovery to normal levels of oxidative markers after treatment [126]. Liver function enzymes and histological pictures of the liver were significantly impacted. Because of the significant improvements in hepatic oxidative markers, it has been reasonable to assume that the extracts provide protection against *Plasmodium* infection [128].

#### 3.2.9. Antidiabetic

Diabetes mellitus is the most common endocrine illness in the world, affecting an estimated 200 million individuals. In 2030, the population is expected to reach 366 million [158]. The highest levels of activity at 25.59 and 39.48%, respectively, at *p* > 0.001 were seen after 7 and 15 days of treatment with 500 mg/kg. At 29.07 and 35.56% after 7 and 15 days, the 500 mg/kg treatment provided the highest hypoglycaemic impact [44]. The extract inhibited alpha-amylase and glucosidase by 54 and 43%, respectively, at doses of 100 g/mL [129]. Diabetic rats exhibited much lower glucose levels and significantly higher blood insulin levels than the control group. The treatment group showed a significant reduction in triglycerides when compared with diabetic control and nondiabetic control rats, showing that it had a hypolipidemic effect (Table 1). The enzyme had high activity against alpha-amylase and glucosidase in methanolic extract, with 8.9 and 39.12 g/mL, respectively [50]. All preliminary research on the plant extract's ability to inhibit alpha-glucosidase and alpha-amylase showed the species has been shown to be a promising antidiabetic source in both in vitro and in vivo studies (Table 1). Diabetes-related disorders may benefit from the plant extract's preventive and therapeutic properties. Clinical trials are important because the investigations on this plant were done *in vitro* and *in vivo*.

#### 3.2.10. Antiobesity

At pandemic levels, obesity is a key factor in the worldwide burden of chronic disease and disability. There are currently over one billion overweight adults in the globe, with at least 300 million of those individuals being classified as clinically obese [159]. Obesity management and therapy necessitates further research on medicinal plants, given the present conditions. In hypercholesterolemic male rats, the extract improved liver and kidney functions and reduced lipid peroxidation. The antihyperlipidemic actions of this extract may be connected with a suppression of oxidative stress because of its high content of phenolic compounds (Table 1).

#### 3.2.11. Antianxiety

Anxiety is a medical condition that affects both our mental and physical health, and it has a variety of characteristics, including cognitive, emotional, behavioural, and somatic. About one-eighth of the global population is affected by anxiety, making it an essential research topic [160]. A number of studies show *Z. spina* to have a significant effect on anxiety (Table 1). Compared with the induction group, the data show that the extract considerably suppresses the expression of the markers investigated. The extract may help protect males from the harmful effects of pentylenetetrazole (Table 1). Administering the extract after HgCl_2_ exposure stopped mercury build-up in the cortical slices. As a result, the levels of malondialdehyde were reduced, as were those of nitrite and nitrate production and nitrite and nitrate creation enzymes. Glutathione levels were also boosted, as were those linked to the antioxidant enzymes glutathione reductase and glutathione peroxidase. Hence, the extract might be used to reduce the damage to neurons caused by HgCl_2_ poisoning. [133].

#### 3.2.12. Anticancer

Cancer is a disease in which cells divide improperly and uncontrolled. In 2012, around 14 million new cancer cases were reported worldwide, with 8.2 million cancer-related deaths [161]. The development and spread of the contemporary healthcare system has been supported by medicinal plants. As their acceptability and acknowledgment spread over the world, medicinal plants remain the only path ahead. According to the findings of this investigation, the leaf extract contains compounds that have anticancer properties, making it a promising target for future research to create novel anticancer medications (Table 1). If extensive scientific research is conducted, the leaf extract of *Z. spina* will aid in the development of novel anticancer drugs. There are certain drawbacks to utilizing natural alternatives to pharmaceutical medications. They can be quite poisonous if they are not properly selected and prepared.

#### 3.2.13. Toxicity

The increased interest in using plant extracts to treat human and animal diseases contributes to the current state of knowledge regarding the use of plant products in medicine. There are certain drawbacks to utilizing natural alternatives to pharmaceutical medications. They can be quite poisonous if they are not properly selected and prepared. Because of this, it is essential to determine the plant extracts' safety. Many studies have proven that medicinal plants contain a wide array of compounds that have a positive biological effect [21, 151]. These components are only beneficial if they are confirmed to be nontoxic or have minimal toxicity. Quite a number of studies have been carried out on the toxicity of *Z. spina* parts (Table 1) both *in vivo* and *in vitro*. In both the short- and long-term experiments, all rats survived at a limit dose of 3000 mg/kg of the root extract. There was no mortality; however, the rats in all groups showed symptoms of tiredness for about 1 to 2 hours (Table 1). Prophage induction did not increase at concentrations of 5, 15, or 30 mg/mL of the leaf extract compared with the control. The pfu/mL did not rise due to the phage's spontaneous release from lysogenic strains, according to the mutagenic index. As a result, no genotoxic potential was found in the plant extracts tested [64]. The leaf extract was discovered to contain a toxic level of 4050 mg/kg BW. When fed at doses below 1500 mg/kg BW, the leaf extract has no toxic effects on the liver (Table 1). Excessive use of the leaf extract may have toxicological consequences, according to the findings of this study; hence, it is recommended that only modest amounts be used.

### 3.3. Chemical Composition

**Figure 3:**
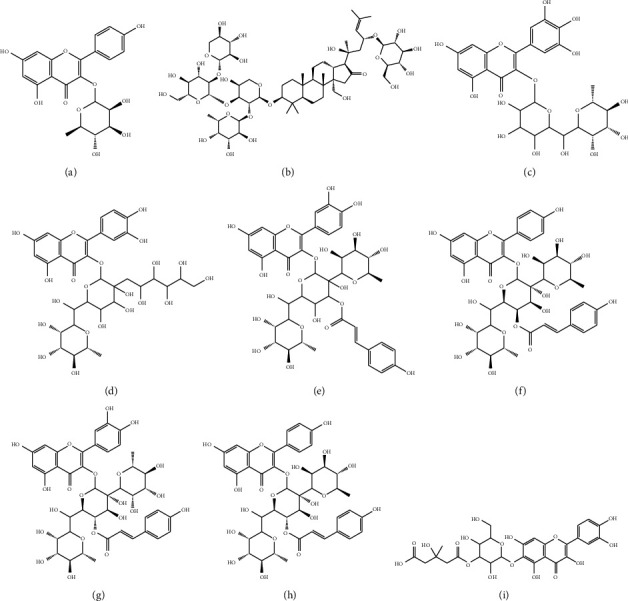
Some of the major compounds from *Z. spina* parts. (a) Kaempferol-3-*O*-rhamnoside. (b) Jujuboside B1. (c) Myricetin-3-*O*-(6-rhamnosyl) hexoside. (d) Quercetin-3-*O*-[(2-hexonyl)-6-rhamnosyl]-hexoside. (e) Quercetin-3-O-p-coumaroyl (2,6-dirhamnosyl)-hexoside. (f) Kaempferol-3-*O*-(4-*O*-(p)-coumaroyl)-2-rhamnosyl-[6-rhamnosyl]-galactoside. (g) Quercetin-3-*O*-(4-*O*-*p*-coumaroyl)-2-rhamnosyl-[6-rhamnosyl]-glucoside. (h) Kaempferol-3-O-(4-O-p-coumaroyl)-2-rhamnosyl-[6-rhamnosyl]-glucoside. (i) Quercetin 3-O-[4-carboxy-3-hydroxy-3-methylbutanoyl]-(6)-hexoside.

Generally, plants have been documented overtime as medicine and/or lead compound sources [162]. Although chemical and synthetic methods have made medicines easier to obtain, the incorporation of plants as sources of medicine or lead compounds in drug discovery has led to the introduction of new and promising lead compounds possessing eccentric biological activities on various diseases [162]. The remarkable biological activity and the traditional medical applications of *Z. spina* have prompted a lot of investigations into its chemical composition. A total of 431 compounds were reportedly isolated from its genus (*Ziziphus*) with alkaloids and flavonoids being reported as the major classes of compounds [163]. In *Z. spina*, saponins, fatty acids, and phenolics in addition to alkaloids and flavonoids have been reported from various parts. This section of this review provides a comprehensive analysis of the phytochemical composition reported from different parts of *Z. spina*. Different parts of *Z. spina* have been reported in several studies to contain diverse classes of phytochemicals. The leaves obtained from Indonesia [29, 83] as well as other parts of the world [70, 85], were reported to have shown the presence of flavonoids, alkaloids, saponins, tannins, steroids, and triterpenes. The presence of monosaccharides, reducing sugars, pentose, ketosis, deoxy sugars, and indole alkaloids were reported in the leaves collected from the Plateau state, Nigeria [122, 164]. The detection of glycosides in the aerial parts and cardiac glycoside in the leaves was also reported [85]. Preliminary phytochemical screening of the fruit, pulp, seeds, and almonds of *Z. spina* collected from Settat and Khouribga cities in Morocco revealed the presence of alkaloids, saponins, triterpenes, quinones, and steroids in the fruit. Alkaloid was reportedly absent in the seed and almonds, while steroid was reportedly absent in the pulp [165]. In contrast to these reports, alkaloids and cardiac glycosides were reportedly absent in the leaves collected from Niger state, Nigeria [93]. Difference in geographical location has been noted to influence the biosynthesis and accumulation of secondary metabolites in plants. In the aerial parts collected in Tabuk, Saudi Arabia [122], and fruits and seeds collected in Qena city, Egypt [75], saponin was reportedly absent. Studies on the bark, fruit, root, seed, and leaf extracts were reported to have shown the presence of steroids, flavonoids, tannins, and alkaloids, but absence of triterpenes [75]. In the same study, phlobatannin was reportedly detected only in the bark extract of the plant. Cyclopeptide alkaloids basically are compounds that are polyamidic in structure with 13-/14- or 15-member ring structure having a side chain that is either basic or neutral in terms of characteristics based on the absence or presence of a terminal nitrogen [163]. These cyclopeptide alkaloids have been reported to be widely distributed in the family Rhamnaceae, particularly the genus *Ziziphus* [166]. Tuenter et al. [167] reported the isolation of two integerrine-type cyclopeptide alkaloids—nummularine-E and nummularine-D—and three amphibine-B 5(14)-type cyclopeptide alkaloids coupled with *β*-hydroxy-proline moiety—spinanine-B, spinanine-C, and amphibine-D—from the stem bark of dichloromethane fraction of *Z. spina*. In another study, a 14-membered cyclopeptide alkaloid, spinanine-A, belonging to amphibian F type was also isolated from the stem bark [168]. From the leaf (80% methanol) extract, the isolation of a new cyclopeptide alkaloid named 4(13) nummularine-C was reported [2]. Many other cyclopeptide alkaloids have been isolated and characterized from different parts of *Z. spina*, and they are as presented in Table 2 and Figure 3. The high content of saponins in the leaves of *Z. spina* that has found commercial use in the making of shampoo and detergents [172] has attracted lots of attention to the phytochemistry of this plant. Using 1D, 2D, HRESIMS, and GC-MS (identification of sugar moieties), identification and characterization of dammarane-type saponins, jujuboside B1, 22*α*-acetoxy christinin A, christinin A_1_, christinin A_2_, lotoside III, and 15-acetoxy lotoside IV were reported from n-butanol fraction of the leaves [172]. Ziziphine-F, jubarine-A, and amphibine-H have been reportedly isolated from the stem bark [168]. A novel triterpenic acid, 13-dehydrobetulin, isolated from chloroform fraction, stem ethanolic extract [20], and 3 new dammarane triterpenoids, sidrigenin, konarigenin, and siconigenin, isolated from the leaves [172] have all been reported in *Z. spina*. Other triterpenic acids that have been reported from *Z. spina* are summarized in Table 2 and Figure 3. The UHPLC/PDA/ESI-MS analytical technique was used to identify and characterize four new *O*-flavonoids—myricetin-3-*O*-(6-rhamnosyl)-hexoside, kaempferol-3-*O*-(2,6-diharmnosyl)-hexoside, kaempferol-3-*O*-rhamnoside, and quercetin-3-*O*-[(2-hexonyl)-6-rhamnosyl]-hexoside—and six new acyl flavonoids—quercetin-3-O-p-coumaroyl (2,6-dirhamnosyl)-hexoside, 6′-caffeoyl 3′,5′-di-C-glucopyranosylphloretin, kaempferol-3-*O*-(4-*O*-*p*-coumaroyl)-2-rhamnosyl-[6-rhamnosyl]-galactoside, quercetin-3-*O*-(4-*O*-*p*-coumaroyl)-2-rhamnosyl-[6-rhamnosyl]-glucoside, kaempferol-3-O-(4-O-p-coumaroyl)-2-rhamnosyl-[6-rhamnosyl]-glucoside, and quercetin 3-O-[4-carboxy-3-hydroxy-3-methylbutanoyl]-(⟶6)-hexoside—isolated from the leaf methanol extract of *Ziziphus spina-christi* [2]. One new polyphenol, quercetin 3-*O*-(4-*O*-trans-*p*-coumaroyl)-*α*-L-rhamnopyranosyl-(1⟶2)-[*α*-L-rhamnopyranosyl-(1⟶6)]-*β*-D-galactopyranoside, was reportedly isolated from n-butanol fraction of the leaves [172]. LC-MS-ESI analysis of the root ethanol extract revealed the presence of epicatechin [41]. Spectra data from HPLC and UV-visible analysis revealed the presence of catechin, gallic acid, ellagic acid, chlorogenic acid, rutin, isoquercitrin, quercetin, and kaempferol in the methanolic extract of the fruit [31]. Moreover, from the fruit were isolated and characterized different flavonoids carrying different sugar moieties. These flavonoids include quercetin 3-*O*-robinobioside, quercetin 3-*O*-*β*-D-xylosyl-(1⟶2)-*α*-L-rhamnoside, quercetin 3-*O*-*β*-D-xylosyl-(1⟶2)-*α*-L-rhamnoside-4^`^-O-*α*-L-rhamnoside, quercetin 3-*O*-*β*-D-galactoside, and quercetin 3-*O*-*β*-D-glucoside [173]. A summary of other polyphenolic compounds isolated/detected in *Z. spina* are presented in Table 2 and Figure 3. Using head space solid-phase microextraction (HS-SPME) and GC-MS methods, identification of *α*-pinene, *β*-pinene, *β*-myrcene, *β*-phellandrene, L-menthone, carane, trans-caryophyllene, and bicyclogermacrene was achieved as the major constituents of the essential oils of *Ziziphus spina-christi* [180]. Odeh et al. [181] reported the presence of benzaldehyde, phenylacetyldehyde, phenylethylalcohol, benzene, acetonitrile, 2-ethyl hexanoic acid, octanoic acid, 2-methoxy-4-(1-propanol)-6-acetate phenol, nonanoic acid, decanoic acid, 1-hydroxy-2,4,6-trimethylbenzene, and 5-hydroxymethyl-2-furan carbonyldehyde in honey obtained from *Z. spina* using HS-SPME-GC-MS methods. In the same study, phenylacetonitrile and 5-hydroxymethyl-2-furan carbonyldehyde were identified as potential markers of honey based on the premise that the different ratios of components present in honey could be utilized as a characteristic to differentiate their floral origins. Said et al. [182] reported the presence of various volatile compounds in the fruits including hexanoic acid, octanoic acid, dodecanoic acid, tetradecanoic acid methyl ester, tetradecanoic acid, hexadecenoic acid methyl ester, oleic acid methyl ester, and oleic acid ethyl ester as the major constituents. GC-MS analysis of stem bark diethyl ether extract after ethyl acetate was reported to have shown the presence of butyl hydroxy toluene bicyclo(4,4,0)dec-2-ene-4-ol, 2-methyl-9-(prop-1-en-3-ol-2-yl), dotriacontane, phytol, and 14-a-H-pregna as the major constituents [66]. Table 2 shows the major constituents of volatile compounds reported from *Z. spina*. Other compounds such as dodecaacetyl prodelphinidin B3, acetyl betulinic acid, sitosterol-tetraacetyl-*β*-D-glucoside, pentaacetyl glucose, and octaacetyl sucrose [174] have all been reported from the leaves. NMR (1D and 2D) and GC-MS were used for the identification and characterization of two cyclic amino acids, namely, 4-hydroxymethyl-1-methyl pyrrolidine-2-carboxylic acid and 4-hydroxymethyl-4-hydroxymehtyl-1-methylpyrolidine-2-carboxylic acid, from the seeds of *Z. spina*. Quantitative phytochemical analysis on different parts of *Z. spina* has been studied. It is noteworthy that different parts of *Z. spina* accumulated different constituents with variations in their quantities.  Table 3 presents the quantitative phytochemical analysis reported on *Z. spina*.

## 4. Conclusion and Future Research

This review attempts to summarize the key findings of numerous research groups involved in the hunt for naturally occurring active principles from *Z. spina* against a variety of human diseases, the objective of which has been to get new effective medications. In this review, the ethnobotanical, pharmacological, and chemical content of the abundantly diversified species have been emphasized. The study found traditionally the plant parts especially the leaves were used for the treatment of diabetes, malaria, digestive issues, typhoid, liver complaints, weakness, skin infections, urinary disorders, obesity, diarrhoea, and sleeplessness. Preclinical investigations have already been conducted on a variety of biological activities. The leaves were found to have significant biological activity, and this is due to the presence of high contents of polyphenol compounds. There is a lot of room for fresh scientific and developmental research. The evidence reported in this analysis provides a foundation for future investigations that are desperately required. Detailed examinations of the following topics are among the top objectives for future research: (1) identification of species based on micromorphology and anatomy in each region of the world, (2) mechanism of action of the isolated compounds, (3) clinical trial, (4) the standard dose and safety of the leaf be established, and (5) formulation of herbal medicine from the leaf.

## Figures and Tables

**Table 1:** Biological evaluation of *Z. spina*.

S/N	Biological evaluation	Method	Solvents	Plant part	Major findings	Reference
1	Antioxidant		Callus extract	Zinc and selenium oxide nanoparticles	On 1-BJ1 normal cells, ZnONPs and SeONPs have promising antioxidant potential	[24]
		DPPH and *β*-carotene-linoleic acid	*n*-hexane	Fruits	With IC_50_ values of 5.5 and 4.1 *μ*g/mL, the fruit extract showed antioxidant activity	[25]
		DPPH, FRAP	Ethanol, hexane		Ethanolic extract demonstrates higher inhibitory activity compared with the hexane. The lower the value, the better the plants' ability to scavenge free radicals	[26]
		DPPH, FRAP	Methanolic, ethanolic, and aqueous	Leaves	With IC_50_ values of 21.4, 24.2, and 54.3 g/mL for methanolic, aqueous, and ethanolic leaf extracts, respectively, the activity demonstrated good antioxidant capacity in terms of radical scavenging activity. The leaf extracts' reducing power was discovered to be concentration dependent	[27]
		*In vivo*	70% ethanol	Leaves	Enhanced balance and motor coordination. Short step-through latency was lengthened when the leaf extract was administered to ischemia rats	[28]
					In addition to reducing malondialdehyde levels in the brain and serum, the leaf extract also increased serum and brain antioxidant ability	
		DPPH	Ethanol	Leaves	With an IC_50_ of 23.4, leaves had a significant activity against free radicals	[29]
		DPPH	Aqueous and ethanolic	Leaves and bark	The activity of the leaf aqueous and ethanolic extracts was 30 and 91 at a concentration of 0.5 mL, respectively, while that of the aqueous and ethanolic extract of the bark was 44 and 70, respectively	[30]
		*In vivo*	70% methanol	Fruits	The findings of this study show that the fruit extract may prevent the development of chronic experimental colitis in rats	[31]
		*In vivo*	Ethanolic, aqueous	Leaves	The leaf extracts had immunologic and antioxidant effects on rabbits exposed to a 2% H_2_O_2_ solution to generate oxidative stress	[32]
		DPPH, FRAP		Seeds	6–12–24–48 h were all used in the fermentation process. Fermented seed extracts had substantially higher phenolic, vitamin C, and total carotenoid contents and antioxidant activity than unfermented samples at *p* < 0.05	[33]
		DPPH	Methanol	Leaves	The leaf extract has an IC_50_ of 33.91 mg/mL for scavenging activity	[34]
		DPPH	Methanol	Leaves	The leaf extract had a high level of antioxidant activity at 0.086 *μ*g/mL	[35]
		DPPH, ribosomal degradation assay	Aqueous	Essential oil	Scavenging activity of the essential was found with an IC_50_ value of 53 ± 2	[36]
		DPPH	Ethyl acetate	Whole plant	The whole plant extract demonstrated significant inhibition capacity at 61 ± 0.04	[37]
		DPPH	Distilled water, methanol	Leaves	A dose-dependent inhibition was seen in distilled water and methanol leaf extract. However, the proportion of free radical inhibition in the n-butanol fraction was greater than that in the other fractions	[38]
		ABTS, DPPH, FRAP, SRSA, TRPA	Methanol	Fruits	All methods exhibited a strong activity, with chelating methods having the highest at 94% at the concentration of 100 *μ*g/mL	[39]
		Rancimat, DPPH		Leaves	The results show that leaf polyphenols, when added to the test system in varying amounts, have antioxidant activity. Similarly, after only 10 minutes, a scavenging capacity of 40.00 was achieved using four different concentrations of phenolic compounds. After the first 10 minutes of incubation, the scavenging capacity remained the same in all cases	[40]
		DPPH, ABTS, and Fe^2+^ chelating assays	Aqueous, methanol, ethanol, acetone	Root	DPPH, ABTS, and Fe^2+^ chelating assays with IC_50_ values of 0.41 ± 0.01; 0.33 ± 0.14; and 0.24 ± 0.03, respectively	[41]
		Peroxidase, catalase assay	Aqueous, ethanolic	Leaves	Leaf extract exhibited a significant activity against the free radicals	[42]
		*In vivo*		Leaves	Antioxidants such as T-AOC, GSH-Px, T-SOD, and CAT considerably greater in the blood of rats fed with high leaf diets	[43]
		DPPH, ABTS	Aqueous, methanol	Leaves	When compared with the benchmark of 100 *μ*g/mL, the scavenging activity of the leaf extract was 96%. With an increase in the concentration, there is a simultaneous rise in scavenging activity	[44]
		DPPH	70% ethanol	Seeds and fruits	The fruits were found to have the highest inhibition of 54.10 at the concentration of 200 *μ*g/mL	[45]

2	Anti-inflammatory	*In vivo*	Ethanol	Root or bark	With a total inhibition of 79.2%. This explains why these plants have long been used as polyherbs to cure ulcers	[46]
		*In vivo*			In addition to reducing ulcer size and reducing colitis indicators, pretreatment with the extract (100, 200, and 400 mg/kg/day) at various dosages slowed the progression of inflammation and prevented mucosal damage. In comparison with the reference medicine, mesalazine (MLZ), ZFE (400 mg/kg) therapy reduced inflammatory colonic damage more significantly	[47]
		*In vivo*		Leaves	Substantially and dose-dependently reduced sepsis-induced liver and spleen damage, according to our findings. These findings imply that, through eliciting anti-inflammatory and antioxidant effects, they could be used to treat sepsis	[48]
		Protein denaturation	70% ethanol	Seeds and fruits	Both portions of the plant extract had anti-inflammatory activity that was comparable to that of the common anti-inflammatory medication diclofenac	[45]
			Methanol	Root (ZS-Ag-NPs)	It effectively enhanced mRNA expression levels of vascular endothelial cell growth factor and decreased oxidative stress as well as vascular cell inflammation in adipocyte CM. Obesity progression and metabolic inflammatory pathogenesis associated with age were successfully decreased by ZS-Ag-NPs' molecular mechanical activity	[49]
		Protein denaturation	Methanol, ethanol	Leaves	High activity was recorded with the methanolic extract even at 95 compared with the standard at 20.2%, respectively	[50]
			Ethanolic, aqueous	Leaves	The ethanolic extract demonstrated significant efficacy as well as modest antipyretic effect	[51]
		*In vivo*	Hexane, chloroform, ethyl acetate, and methanol	Root	In all models, except the tail-flick test, where the activity was not statistically significant, the percentage exhibits some amount of dose-related effect	[52]
		*In vivo*		Leaves (AgNPs)	Pretreatment with nanoparticles improved histological parameters such as little infiltration and fibrosis, low pleomorphism, and reduced hepatocytes and degeneration	[53]
		*In vivo*		Leaf	Using extract ointments for burns is beneficial	[54]
					Healing with histological alterations, however, the group treated with leaf extract had the greatest improvement	
					Ointment had a better cellular response to the inflammatory process than the other group in which re-epithelialization appears early in the healing process	

3	Antibacterial		Ethanol, aqueous	Leaves		[55]
		Agar diffusion	Ethanol, petroleum ether, ethyl acetate, methanol, and aqueous	Leaves, stem bark, fruits	The extract at the concentration of 100 mg/mL was found to have activity against some of the tested strain with methanolic extract having a minimum MIC at 6.25 *μ*g/mL. These findings offer promising preliminary evidence for the use of crude extracts in the treatment of bacterial infections	[56]
		Disc	Ethanol	Leaves	The highest zone of inhibition was found at 15 mm against *E. coli*	[57]
		Cup-plate agar diffusion	Petroleum ether, chloroform, methanol, and aqueous	Fruits, leaves, seeds, and stems	Methanol extracts from all parts had the highest activity, followed by chloroform and petroleum ether. No activity was recorded from aqueous extracts	[58]
			Ethanolic, aqueous	Leaves	It also stopped the tested strain from growing. However, there was no evidence of analgesic or diuretic action. Surface activity was seen in the aqueous extract of the leaves, with a threshold micelle concentration of 0.25 percent w/v	[51]
		Agar well diffusion		Leaves	With an MIC value of 0.25 mg/mL, *Escherichia coli* was found to be the most vulnerable bacterium to the extract, while *Staphylococcus aureus* was discovered to be the most resistant strain with an MIC value of 1.00 mg/mL. To summarize, the plant's leaves might be used in food processing and further investigated for the treatment of microbial illnesses	[59]
		Disc diffusion	Aqueous	Seeds	The extract exhibited substantial action against all tested MDR strains. Besides, its polyphenol component demonstrated a stronger impact. Furthermore, the entire extract MIC varied between 3.125 and 12.5 mg/mL and MBC was 3.125–25 mg/mL against prior strains. While the polyphenol fraction, MIC and MBC were around 0.312–1.25 mg/mL and 0.312–2.5 mg/mL, respectively	[60]
			Aqueous, ethanolic	Leaves	Both extracts have an inhibitory effect on many bacterial species in this investigation. Most effective at 200 mg/mL when they came to killing germs	[61]
			Callus extract	Zinc and selenium oxide nanoparticles	The results showed that both crystals have antibacterial ability against the tested strains, with SeONPs having stronger antimicrobial activity than ZnONPs	[24]
		Agar well	Hexane	Seed oil	At 11, 10, 8, and 8 mm, it was active against the tested strains. The findings of this study have established scientific validity for the use of this seed oil in herbal medicine to treat bacteria-related diseases	[62]
		Disc diffusion	Ethanolic and methanolic	Leaves	Both extracts were discovered to be potent antibacterial agents against all microorganisms tested. At concentrations of 128, 100, 64, and 32 mg/L, inhibitory action was measured. At 128 mg/L, the ethanolic extract exhibited the maximum activity of 21 mm against *Salmonella* sp., whereas the methanolic extract had the lowest activity of 9 mm against *Escherichia coli*	[63]
		Disc diffusion	Ethanol	Leave	At doses of 5, 15, and 30 mg/mL, the average diameter of the inhibitory zone was 0 to 17 mm. At 30 mg/mL, it is highly effective against all used bacterial strains. With *Staphylococcus aureus*, the largest inhibitory zone diameter was 17 mm	[64]
		Agar diffusion	Aqueous and ethanolic	Leaves	The inhibitory zone's diameter in cm varied between 1.5 and 2.3 and 2 and 2.2, respectively	[65]
		Agar well	Ethanol	Stem bark	At *p* < 0.05, there was a notable increase in activity. Gram-positive bacteria were more vulnerable to this extract than gram-negative bacteria, according to these findings	[66]
		Well-diffusion method	Aqueous	Honey	The microbiological strains were severely hampered. There were no resistant microbial strains, with the highest sensitivity at 36 mm	[67]
		MIC	*n*-hexane	Fruits	The minimum inhibitory concentrations against the tested microorganisms ranged from 32 to 125 g/mL	[25]
		Agar-well diffusion, MIC, MBC	Methanol	Fruits	Overall, the research found that the fruit extract possessed Gram-negative bacteria that have no antibacterial action, while moderate antibacterial activity was shown against gram-positive bacteria. The discovered actions, however, were not noteworthy compared with antibiotics	[68]
		Well-diffusion method	Aqueous	Aquatic leaves, aquatic stem bark, and combination of leaves + stem bark	All of the tested strains were extremely sensitive to a combination of leaves and stem bark within the inhibition zone of 25–35 mm	[69]
		Disc diffusion	Ethanol, aqueous	Leaves	The ethanolic leaf extract had the highest activity against *S. aureus* at 18 mm, while the aqueous extract had the lowest activity against *B. subtilis* at 13 mm	[70]
		Disc diffusion method	Ethanol	Leaves	Within the 8–26 mm range, there was a lot of activity	[71]
		Agar well	AgNO_3_ aqueous	Leaf	The SNPs had good activity against *S. aureus* and *E. coli*, with inhibition zones of 18 and 20 mm, respectively	[72]
			Methanol	Leaves	The extract revealed activity via secondary metabolites such as alkaloids and flavonoids. Significant influences on microbial growth harmed energy metabolism, leading to fat accumulation and protein inhibition	[73]
		Well diffusion method	Methanol	Leaves, fruits, and stems	Leaf extract exhibited higher inhibition zone at *p* < 0.001	[74]
		Agar well	Ethanol and methanol	Bark, leaves, fruits, seeds and roots	All of the bacterial strain tested were sensitive to plant extracts. Except for *Enterobacter aerogenes*, the bark extract was the most effective against all bacteria	[75]
		Well diffusion	Aqueous and ethanol	Leaves and stem bark	The aqueous stem bark extracts had inhibition on the tested strains with the highest inhibition on *Klebsiella* spp. and *E. coli* at 20 mm, respectively	[76]
		Disc diffusion	Aqueous	Leaf (AgNPs)	The extract had a good inhibitory impact on all gram-positive and gram-negative bacteria tested, with the maximum activity against *P. aeruginosa* 16 mm	[77]
			Ethanol		It was found to exhibited activity at 9, 6, 7, 5, and 6 mm against the tested strain	[78]
		Well diffusion	Aqueous	Leaf (AgNO_3_)	Maximal inhibitory zones activity of 24, 23, 15, and 17 mm in the extract, respectively	[79]
		Agar disc diffusion	Ethanolic	Seed	These extracts demonstrated inhibitory activity at various stages of germination; the first stage had 22 mm inhibitory activity against *S. faecalis* and 20 mm against *S. aureus*, and 15 mm inhibitory activity against *P. aeruginosa*. The second stage exhibits 15 mm against *S. faecalis*, 14 mm against *P. aeruginosa*, and 10 mm against *S. aureus*	[80]
		Plate agar method	Petroleum ether, chloroform, 80% ethanol, and aqueous	Leaves, fruits, and seeds	A significant activity was recorded from the extract	[81]
		Agar diffusion	Methanolic	Leaves	*B. cereus* 15, *C. perfringens* 12, *L. monocytogenes* 11, *S. aureus* 10, *P. vulgaris* 8.5, and *V. parahaemolyticus* 8 mm, respectively, were tested. The extract had no discernible effect on the remaining strains tested	[82]
				Leaves	With a probability activity value of more than 0.300, PASS analysis revealed that 15 compounds (64.51 percent) have antibacterial potential. The extract inhibited pathogenic bacterial growth in a moderate-to-strong manner, except for *V. vulnificus*, for which it provided a poor inhibition	[83]
		Disc diffusion	Ethanol, methanol	Leaves	Both extracts had the lowest antibiofilm impact on the tested strains	[84]
		Disc diffusion	Aqueous, ethanol	Leaves	On all of the studied strains, the MIC shows that the aqueous extract ranges from 12.8 to 8.3 mg/mL, whereas the ethanolic extract ranges from 13.5 to 8.8 mg/mL	[85]
		Agar well diffusion	Aqueous	Leaves	The extracts at 50 *μ*g/mL had no effect on any of the bacterial strains, while the greatest activity was at 100 *μ*g/mL with 9 mm zone of inhibition against *Klebsiella oxytoca* and *Proteus mirabilis*, respectively	[86]
		Diffusion assay		Fruits	This lipid fraction was active against the tested bacterial strains. At 2.6 mm, the fatty acid fraction had a lot of activity against it	[87]
		Disc diffusion	Methanol	Leaves, stem	At the concentration of 200 mg/mL recorded an inhibition zones of 16, 14, and 16 mm, respectively	[88]
		Agar well	Aqueous and methanolic	Leaves and seeds	Against five bacterial strains at varied doses of 25, 50, 100, and 200 mg/mL, respectively. At 25 mg/mL, no activity was recorded. The leaves aqueous had the maximum activity at 17.67 mm against *Staphylococcus aureus* and, similarly, had the lowest activity at 7.33 mm against *Pseudomonas aeruginosa*	[89]
		Disc diffusion	Ethanol, petroleum ether, ethyl acetate	Leaf, seed, young stem, fruits, and root	It has moderate activity with the minimum inhibition at 6 and maximum at 10 mm, respectively	[90]
		Disc diffusion	Aqueous and ethanolic	Leaves and bark	Ethanolic bark inhibits higher inhibition with at 22 for *Escherichia coli* and 15 mm for *Staphylococcus aureus*, respectively	[30]
		MIC, MBC	Ethanol	Leaves	Have minimum inhibitory zone activity against bacteria, enteropathogenic *E. coli* at concentrations of 50% and minimum bactericidal activity at concentrations of 75% at 106 CFU/mL	[91]
		Agar well	Ethanol	Leaves	At a dosage of ≥0.25 g/mL, it has antibacterial action and inhibits the bacteria *P. acne*	[29]
		Disc diffusion	Ethanol, methanolic	Leaves	The maximum inhibitory activity for *S. aureus* and *B. cereus* were 18 and 14 mm for methanolic extract and 15 mm for ethanolic extract against *S. aureus* and *P. mirabilis*, respectively	[92]
		Agar cup	Ethanolic	Leaves	MIC and MBC were 20 mg and 40 mg mL^−1^, respectively	[93]
		Well method				
		Agar well	Aqueous, ethanol	Leaves	The greatest rate of inhibition diameter against *Staphylococcus aureus*, 18 mm in concentration 500 mg mL. No activity was recorded against the tested strains from aqueous extract	[94]
		Agar plate diffusion and broth dilution	Aqueous	Stem bark	The extract showed efficacy against all organisms tested. Except for *S. pyogenes*, which had an MIC of 25 mg/mL, the MIC was 12.5 mg/mL against all species	[95]
		Agar well	Methanol and ethanol	Leaves	The activity of 2.5 percent NaOCl against *E. faecalis* was the strongest, followed by hydroalcoholic and methanolic extracts. Until research like this identifies a better alternative, NaOCl is an effective irrigant in root treatment	[96]
		In vitro, in vivo	Aqueous cold water and ethanol	Leaves	Significantly inhibited the growth of the tested strains at 95%	[97]
		Agar well	Aqueous ethanol	Bark	The maximum inhibition was recorded at 22 mm against *E. coli*	[98]
		Microtiter plate	Hot and aqueous	Leaves	With the concentration of 50 mg/mL, the results demonstrated the ability of the extract to prevent biofilm formation	[99]
		Disc diffusion		Leaves	Any inhibition below 6 mm was considered as no activity with 800 *μ*g/disc. The following study found no activity against the tested strains	[100]
		Agar well	Aqueous, ethanolic	Leaves	Ethanolic leaf extract exhibited the highest activity against *S. aureus* with an inhibition zone of 20 mm	[101]
		Cup-plate agar diffusion	Petroleum ether, ethyl acetate, ethanol, methanol, and distilled water	Stem bark	Studies revealed that the methanolic extract at 100 mg/mL exhibited the highest activity of 25 mm. The extract reduced the development of all bacteria, with most extracts showing antimicrobial action on multiple levels	[102]
		Disc		Honey	Demonstrate a significant activity against the tested strain	[103]
			Aqueous	Leaves	*S. aureus* and *S. haemolyticus* biofilm formation was prevented by a hot extract tested against the biofilm formation. The results showed that the two antibiotics and the plant extract could both prevent the biofilm formation	[104]
		Agar well	Aqueous and ethanolic	Leaves	The aqueous extracts demonstrated a significant inhibition zone of 5.6 mm against *Streptococcus pyogenes* at a dosage of 100 mg/mL, with the least inhibition around *S. aureus*. The ethanolic extract, at a dosage of 100 mg/mL, has the biggest inhibitory zone against *Klebsiella pneumonia*, measuring 6.6 mm. Our findings imply that is a good alternative antibacterial agent against a variety of pathogenic bacteria	[42]
		Pour-plate method		Ziziphus honey	In most dilutions, the extract was higher after 120 hours of incubation for each of the tested strains. After 120 hours, the microbial count was reduced by 3–7.5 logs compared with the control	[105]
		Well diffusion methods	Aqueous	Fruits	AgNPs' inhibitory activity against all investigated human pathogenic microorganisms rose with fruit ripening progress from mature fruit to unripe fruit to immature fruit	[106]
		Cup-plate agar diffusion	Ethyl acetate	Whole plant	At the bacterial concentration of 1 mg/mL, the extract recorded the highest inhibition of 22 mm against *S. aureus*	[37]
		Agar plate	Aqueous	Pulp	Study revealed increased sensitivity to *E. coli* and *P. aeruginosa*. MIC of 6.25 mg/mL. For *S. pyogenes*, MBC of 12.5 mg/mL	[107]
		Micro broth dilution	Aqueous	Essential oil	Inhibited the development of *Penicillium digitatum* and *Klebsiella pneumonia* at doses of 128 and 512 g/mL, respectively	[36]
		Microdilution method	Hot water and ethanol	Leaves	The ethanolic extract has the highest inhibition at 4 ± 1.03	[108]

4	Antifungal	Cup-plate agar diffusion	Petroleum ether, chloroform, methanol, and aqueous	Fruits, leaves, seeds, and stems	No activity recorded	[58]
			Ethanol	Leaves	In comparison with the control, cells treated with 150 *μ*L/mL of the extract had an average sterol content approximately three times higher	[109]
		Cup-plate agar diffusion	Petroleum ether, ethyl acetate, ethanol, methanol, and distilled water	Stem bark	Exhibited strong activity at concentration 100 mg/mL with an inhibition zone of 20 mm. These discoveries can be used to aid in the treatment of fungal illnesses	[102]
		Agar well diffusion	Ethanol and methanol	Leaves	According to the findings, the ethanolic extract possesses antifungal characteristics and can be utilized to treat fungal infections. More research is needed to evaluate the effectiveness of this plant in treating candidiasis patients	[110]
		Micro broth dilution	Aqueous	Essential oil	In a dosage of 64 *μ*g/mL, 99.9% of *Aspergillus niger* does not grow	[36]
		Agar well	Ethanol	Stem bark	Fungal strains with significant activity at *p* < 0.05 ranged from 3.90 to 32.3 g/mL	[66]
		Well diffusion	Aqueous	Honey	The microbiological strains were severely hampered. There were no resistant microbial strains, with the highest sensitivity at 36 mm	[67]
		Agar well	Aqueous, ethanolic	Leaves	At a dosage of 500 mg/mL, both ethanolic and aqueous extracts inhibited *Candida albicans* with inhibition diameters of 32 mm and 18 mm, respectively	[94]
					The study revealed increased sensitivity to the tested strain, indicating the antifungal activity of the extract	[107]
		Agar plate diffusion and broth dilution	Aqueous	Stem bark	There was no inhibition at low doses, and it was susceptible above 600 mg/mL, implying that the extract has antifungal potential	[95]
		Agar well	Aqueous and ethanolic	Bark	The maximum inhibition was recorded at 17 mm	[98]
			Callus extract	Zinc and selenium oxide nanoparticles	Both crystals have antifungal activity against the tested strain, with SeONPs having greater antifungal activity than ZnONPs, according to the findings	[24]
		Agar cup well	Ethanolic	Leaves	No activity against the tested fungal strain	[93]
		Agar dilution	Aqueous	Leaves	The extracts were found to have action against *Fusarium*, *Helminthosporium*, *Alternaria*, and *Rhizoctonia* species, as well as inhibiting *Alternaria* and *Fusarium* sporogenesis	[111]
		*In vivo*	Ethanol	Leaves	A shampoo containing the plant extract was then developed and tested on 80 people with dandruff for a period of four weeks. With the Sidr shampoo formulation, 86% of the participants reported significant improvement in their dandruff symptoms	[112]
		Plate agar method	Petroleum ether, chloroform, 80% ethanol, and aqueous	Leaves, fruits, and seeds	A significant activity was recorded from the extract	[81]
					Extracts were found to have antifungal efficacy against all fungi examined. These findings suggested that the extracts could be used as a substitute for chemical additives in the treatment of fungal diseases in plants	[113]
			Ethanol	Leaves	When treated with the extract at a dosage of 20%, it failed to generate spores	[114]
		96-well plates	Ethanol (80%, v/v)	Fruits (unripe and ripe)	The minimum inhibitory concentration 90 values for ripe and unripe fruits were 25 and 0.1 g/mL, respectively	[115]
		Agar diffusion	Methanolic	Leaves	No activity	[82]

5	Antidiarrhoeal	*In vivo*		Stem bark	The extract was shown to protect rats against castor oil-induced diarrhoea as well as reduce intraluminal fluid collection and gastrointestinal transit, according to the results. In mice, the intraperitoneal and oral LD_50_ values were 3465 and 1200 mg/kg, respectively	[116]

6	Antiparasitic	*In vivo*	70% ethanol	Leaves	Endothelial contraction was dose-dependent and substantial (*p* < 0.0001) in both intact and denuded aortas. A comparable reaction to the leaf extract at a concentration of 5 mg/mL was seen with KCl (50 mM). Aortic endothelium intact and denuded aorta contractions were decreased by 66.7% (mean + SEM) and 71.67% (mean + SEM) when verapamil was applied, respectively	[117]
			Aqueous	Leaves	At concentrations of 6, 25, 12.5, 25, 50, 100, and 200 mg/mL, the drug effective against Egyptian species of schistosomes	[118]
		*In vivo*	70% methanol	Leaves	As a result, the extract was shown to have antiapoptotic, antifibrotic, antioxidant, and protective properties against *S. mansoni*-induced liver lesions in this investigation. The extract's antifibrinogenic and nuclear factor erythroid 2-related factor 2 (Nrf2) advantages against *S. mansoni* were due to the enhancement of these two pathways	[47]
			Aqueous	Leaves	The findings indicated that the extract at concentrations of 500, 250, and 125 *μ*g/mL killed 100% of Egyptian *Schistosoma* strains of adult worms and *schistosomula* of *S. haematobium* within 6 to 12 hours of incubation. As a result, these medicinal plant extracts might be utilized to treat schistosomiasis in a safe and effective manner	[119]
		*In vivo*	70% methanol	Leaves	In the 3^rd^, 4^th^, and 5^th^ groups, oocyst shedding was dramatically reduced to roughly 10.7 × 10^3^, 28.3 × 10^3^, and 23.8 × 10^3^ oocysts/g faeces, respectively	[120]
		*In vitro/in vivo*	Aqueous and methanolic	Leaves	On day 21 after treatment, the extract exhibited a decrease in egg count percent (EPG) in faeces of 61.5 and 78.7% at dosages of 100 mg/kg and 400 mg/kg. EPG of faeces decreased by 24.4, 73.1, and 85.1% at 100, 400, and 800 mg/kg dosages, respectively	[121]
			Methanolic	Leaves	The extract significantly reduced the viability of *L. major amastigotes* at *p* < 0.001, whereas it induced NO production and release, apoptosis, and plasma membrane permeability in macrophage cells with no evidence of harm	[122]
		MTT assay	Methanolic, aqueous		Aqueous extracts had IC_50_ values of 60 and 80 *μ*g/mL, respectively, for methanol and water. Promastigotes of *L. major* were severely affected by all plant extracts	[123]
		*In vivo*	70% methanol	Leaves	*E. papillata* infection enhanced the generated jejunal damage. Furthermore, treatment of infected mice with 100 and 300 mg ZLE/kg increased the number of goblet cells in the jejunal villi substantially	[120]

7	Antiviral	Plate agar method	Petroleum ether, chloroform, 80% ethanol, and aqueous	Leaves, fruits, and seeds	A significant activity was recorded from the extract	[81]
		*In vivo*		Leaves	The leaf extract was effectively used to treat rashes on a 50-year-old man	[124]
		*In vivo*	Ethanol	Leaves	Significantly reduced the growth of the rashes compared with the control group	[125]

8	Antimalarial	*In vivo*	70% methanol	Leaves	The liver and spleen histopathology examinations revealed severe histological abnormalities. The histopathological appearance of the liver and spleen in treated animals improved significantly. Treatment resulted in a significant return of oxidative indicators to normal levels, according to biochemical analyses	[126]
		*In vivo*		Leaves	To sum up, the findings suggest that the extract's antiplasmodial and antioxidant properties might help reduce the devastation caused by *P. berghei*-induced cerebral malaria	[127]
		*In vivo*	70% methanol	Leaf	Had a considerable impact on liver function enzymes as well as histological images of the liver. It is possible to infer that the extracts protect against *Plasmodium* infection, as shown by considerable improvements in hepatic oxidative indicators	[128]

9	Antidiabetic	Alpha amylase and glucosidase assay	Methanol, ethanol	Leaves	The enzyme exhibited strong activity with methanolic extract against alpha-amylase and glucosidase at 8.9 and 39.12 *μ*g/mL, respectively	[50]
		*In vivo*	Methanol	Leaves	At concentrations of 100 *μ*g/mL, the extracts were shown to inhibit alpha-amylase and glucosidase by 54 and 43%, respectively	[129]
		*In vivo*	Ethanol	Leaves	Compared with the control group, diabetic rats had significantly lower glucose levels and significantly higher blood insulin levels. When compared with diabetic control and nondiabetic control rats, the treatment group demonstrated a significant reduction in triglycerides, indicating that it had a hypolipidemic impact	[130]
		*In vivo*	Aqueous and methanolic	Leaves	Following therapy with 500 mg/kg, the highest activity at 25.59 and 39.48% after 7 and 15 days, respectively, was discovered at *p* > 0.001. Similarly, the 500 mg/kg therapy had the greatest (*p* > 0.001) hypoglycaemic effect at 29.07 and 35.56% after 7 and 15 days, respectively	[44]

10	Antiobesity	*In vivo*		Leaves	Improved liver and kidney function and lowered lipid peroxidation in hypercholesterolemic male rats treated with the extract. As a result of its high concentration of phenolic chemicals, the antihyperlipidemic effects of this extract might be linked to inhibition of oxidative stress	[131]
		*In vivo*	Aqueous	Seed	Biochemical and histological changes were reversed with the extract in G3 therapy. The extract lowered hypercholesterolemia, inhibited oxidative stress, and restored biochemical and histological characteristics that had been changed	[132]

11	Antianxiety	*In vivo*		Leaf	Administering the extract after HgCl_2_ exposure stopped mercury build-up in the cortical slices. As a result, the levels of malondialdehyde were reduced, as were those of nitrite and nitrate production and nitrite and nitrate creation enzymes. Glutathione levels were also boosted, as were those linked to the antioxidant enzymes glutathione reductase and glutathione peroxidase. Might be used to reduce the damage to neurons caused by HgCl_2_ poisoning	[133]
					Rotarod testing was utilized to assess motor coordination. The raised plus maze's open-arm duration was dramatically lengthened when *Z. spina* extract (200 mg/kg) was administered. A lower proportion of entrances into the closed arms and less time spent in the closed arms were both lowered by the extract. Motor coordination and balance were unaffected by the concentration of *Z. spina* extract in the study. Scopolamine-induced anxiety is greatly reduced by *Z. spina* hydroalcoholic extract	[134]
		*In vivo*	Ethanol	Leaves	The leaf extract significantly reduces the expression of the indicators studied compared with the induction group. The extract may protect the male against pentylenetetrazole-induced harm	[135]

12	Growth promoter	*In vivo*		Leaves	The findings of this study could have supported the use of low doses of a 20 g SL/kg diet as natural growth promoters without affecting rabbit performance	[136]
					R2 had significantly greater end weight and total growth (*p* < 0.05) than R1, but not significantly higher than R3. R2's daily increase, on the other hand, was much greater than R3's. As a result, feed additives of ZSCL (15 g/kg DM) are strongly suggested in the feeding practices of developing lambs	[137]
		*In vivo*		Leaves	Consumption level of 10%, the findings revealed that the treatment had a significant impact on broiler chicken output and mortality (*p* < 0.05). In the care of broiler chicks, the extract may be used as an alternative to synthetic nutrients	[138]

13	Insecticidal		Aqueous, methanol, ethanolic, and acetonic	Root	The extract has the strongest insecticidal potential when tested against *Lasioderma serricorne*. The ethanolic and acetonic extracts had LC_95_ values of 3.17 and 4.86 *μ*g/insect, respectively. The current study adds to the usefulness of *Z. spina* as a source of epicatechin, which can be employed as a bio-antioxidant and a bioinsecticide	[41]

14	Anticancer	MTT assay	Methanol	Leaves	The IC_50_ of the extract for the RD cell line was 154.44 *μ*g/mL. The extract demonstrated a possible antiproliferative effect against the RD cell	[34]
		MTT assay	70% ethanol	Leaves	On MCF7 cells, extracts had a cytotoxic impact. In MCF7 cells, 1 mg/mL dramatically boosted the expression of the Bax and Bcl-2 genes	[139]
		MTT	Aqueous	Leaves (silver nanoparticles)	Polysaccharides had an IC_50_ value of 1.5 mg/mL, but the IC_50_ value for polysaccharide-coated AgNPs was 0.705 mg/mL	[140]
		MTT	Ethanol, ethanol-aqueous, aqueous	Leaves	After forty-eight hours of administration, the ethanolic fraction had the lowest IC_50_ value of 0.02 mg/mL and triggered cell cycle arrest at the G1/S phase as well as apoptosis	[141]
		*In vivo*	Methanolic	Leaves	Extract treatment of DENA-induced hepatocarcinoma alleviated all except cholangioma-induced abnormalities. Finally, ZSCL (300 mg/kg BM) showed a significant therapeutic effect against DENA-induced hepatocellular cancer by focusing on oxidative stress and oncogenes	[142]
		MTT assay	Hexane	Leaves	According to the findings of this study, the leaf extract contains chemicals with anticancer action, making it a promising target for further research to identify new anticancer drugs	[143]

15	Toxicity	*In vivo*	Aqueous	Stem bark	For the liver enzymes ALT, AST, ALK, serum protein, and albumin, biochemical examination revealed no significant difference between the different concentrations treated at 200, 400, and 800 mg/kg correspondingly and the control group. Furthermore, there was no significant variation in serum electrolytes (*p* > 0.05)	[95]
		Prophage F116 induction	Ethanol	Leaves	When compared with control, the employed concentrations at 5, 15, and 30 mg/mL did not reveal an increase in prophage induction. The mutagenic index revealed that the spontaneous release of phage from the lysogenic strains resulted in no increase in pfu/mL. As a result, the plant extracts evaluated have no genotoxic potential	[64]
		*In vivo*	Ethanol	Root, bark	In both the short- and long-term studies, all rats survived at a limit dose of 3000 mg/kg. There was no mortality, although the rats in all groups showed evidence of sleepiness for about 1 to 2 hours	[46]
		*In vivo*	Aqueous-methanol	Seeds	Lymphocytes, platelets, direct and total bilirubin, albumin, alanine aminotransferase, alkaline phosphatase, aspartate aminotransferase, serum Ca^2+^, creatinine, urea, and organ-body weight ratios were all significantly elevated at *p* < 0.05 by the extract. At 600 and 1000 mg/kg BW, the extract induced hepatic vascular congestion and fibrosis but had no effect on the kidney's histoarchitecture. There are hepatotoxic and nephrotoxic properties in the extract, thusly folklore medicine calls for caution when using this herb	[144]
		MTT assay	Ethanol	Stem bark	In HCT-116 and MCH-7 cell lines at a concentration of 50–400 g, the control cells had a high rate of proliferation, which was taken as 100%	[66]
		1-BJ1 normal cells	Callus extract	Zinc and selenium oxide nanoparticles	Low toxicity was recorded. The particles show potential antibacterial and antioxidant actions and will be used to combat resistant microorganisms	[24]
		Fl-cells	Ethanol		Showed no toxicity	[78]
		*In vivo*	Hydroalcoholic	Leaves	No serious side effect was observed	[125]
		*In vivo*		Leaves	No changes in liver or kidney function were found after the juice was given to the animals. It was found that a dose of BHT (200 parts per million) significantly increased the enzyme activity and serum levels of all three products.	[40]
		*In vivo*	Methanolic	Seeds	Phagocytic index values were the lowest in snails exposed to LC_50_ of the extracts. Results demonstrated no mortality in *Daphnia magna* individuals during the first 12 h of the trial	[145]
		*In vivo*	Aqueous	Leaf (AgNPs)	When compared with MeHg intoxication, AgNPs/MeHg caused a far larger increase in lipid peroxides as a marker of oxidative stress, and of course, compared with healthy control animals	[77]
		*In vivo*	Ethanol	Leaves	The animal model received daily oral dosages of 50 to 200 mg/kg for 28 days. Biochemical tests comparing the extract's toxicity to that of a control group revealed no differences. However, oral administration of the extract at dosages of 100 and 200 mg/kg resulted in minor histologically detrimental effects on liver and kidney tissues	[112]
		*In vivo*	Aqueous	Fruits	All biochemical indicators and histological images of the liver, kidney, and testis improved significantly in animals treated with the extract alone or in combination with AF. The extract may have a powerful function in protecting against aflatoxicosis	[146]
		*In vivo*	Ethanol	Leaves	14-day course of oral 400 mg/kg extract dose. Lactate dehydrogenase and total bilirubin levels in rats' blood were substantially elevated (*p* < 0.05) after oral administration of the extract, but no other liver enzymes were affected. Increased insulin levels, reduced triglyceride levels, and no significant changes in the other lipid profile components were seen when therapy was administered	[147]
		*In vivo*	Aqueous	Pulp	There was no significant difference between the treatment group and the control group in terms of liver enzymes such as ALT, AST, ALK, serum protein, and serum albumin, according to the results of the biochemical study	[107]
			Aqueous, ethanol	Leaves	Both kinds of extracts in concentrations of 500 mg/mL showed no cytotoxicity towards red blood cells	[94]
			Aqueous	Leaves	Extract did not exhibit any morphological alterations from the control at MNTD values of 250, 350, and 300 *μ*L/mL, respectively, in a cytotoxicity experiment	[118]
		*In vivo*		Leaf	In addition, after HgCl_2_ poisoning, a shift in apoptotic proteins in favor of proapoptotic proteins was identified. However, combining the extract with HgCl_2_ considerably reduced the molecular, biochemical, and histological changes caused by HgCl_2_ intoxication. Our results imply that the extract might be utilized to reduce the effects of HgCl_2_ exposure on reproduction	[148]
		*In vivo*	Ethanol	Leaves	The leaf extract has no harmful impact on the liver when administered at dosages below 1500 mg/kg BW. In conclusion, the hazardous dosage of leaf extract in white Wistar rats is over 4000 mg/kg BW	[149]

*Note*. MIC: minimum inhibitory concentration, MBC: minimum bacterial concentration, DPPH: 2,2-diphenyl-1-picrylhydrazyl, FRAP: ferric reducing antioxidant power (FRAP) assay.

**Table 2:** Compounds reported from *Z. spina*.

Compounds	Technique(s)	Quantity (%/*μ*g/g DW)	Plant part (s)	References
Cyclopeptides				
Mauritine F	UHPLC-PDA-ESI-MS		Leaves	[2]
Sanjonine F				
Sanjonine B				
Lotusanine A/frangulanine				
Jubanine C				
Adouetine Z				
Scutianine A				
Oxyphyline A	UHPLC-PDA-ESI-MS		Leaves	[2]
	HPLC-DAD-MS and HPLC-PDA-(HRMS)-SPE-NMR		Stem bark	[167]
Spinanine-A	MS, UV, IR		Stem bark	[169]
Mauritine A	MS, IR, PMR, co-TLC, and optical rotation		Bark	[170]
Mauritine C				
Amphibine F				
Amphibine E				
Amphibine A				
Saponins				
Christinin A	MS, IR, and NMR		Leaves	[171]
Christinin C				
Christinin D				
Christinin B	UHPLC-PDA-ESI-MS/MS, IR, and NMR		Leaves	[2, 171]
Christinin A/C				
Christinin A2	NMR and HRESIMS/UHPLC-PDA-ESI-MS		Leaves	[2, 172]
Jujubogenin-3-O-(di-deoxyhexosyl)-hexoside	UHPLC-PDA-ESI-MS		Leaves	[2]
Jujubasaponin II/III isomer				
Polyphenols				
3′,5′-di-C-*β*-glucosylphloretine	NMR and HRESIMS		Leaves	[172]
	HPLC–PDA–MS and NMR		Fruits	[173]
Hexaacetyl (+)-gallocatechin	NMR		Leaves	[174]
Hexaacetyl (-)-epigallocatechin				
Kaempferol 3-O-robinobioside	NMR and HRESIMS		Leaves	[172]
	HPLC-PDA-MS and NMR		Fruits	[173]
Rutin	HPLC/LC-MS/MS/UV and NMR		Leaves	[29, 48, 175]
Spinosin	HPLC		Leaves	[48]
Ellagic acid				
Isoquercetrin				
Apigenin				
Kaempferol				
Kaempferol 3-O-rutinoside	NMR and HRESIMS		Leaves	[172]
	HPLC-PDA-MS and NMR		Fruits	[173]
Gallocatechin	HPLC		Leaves	[48]
Epigallocatechin	NMR and HRESIMS			[172]
Quercetin 3-O-*α*-arabinosyl-(1⟶2)-*α*-rhamnoside	NMR, HRESIMS/HPLC-PDA-MS, and NMR		Leaves	[172]
			Fruits	[173]
Quercetin 3-O-*β*-xylopyranosyl-(1 ⟶ 2)-*α*-rhamnopyranoside 4ʹ-O-*α*-rhamnopyranoside	NMR and HRESIMS		Leaves	[172]
Quercetin 3-O-*α*-rhamnopyranosyl-(1 ⟶ 6)-*α*-rhamnopyranosyl-(1 ⟶ 2)-*β*-galactopyranoside				
Prodelphinidin				
Quercetin 3-O-robinobioside	HPLC-PDA-MS and NMR		Fruits	[173]
Quercetin 3-O-*β*-D-xylosyl-(1 ⟶ 2)-a-_L_-rhamnoside				
Quercetin 3-O-*β*-D-galactoside				
Quercetin 3-O-*β*-D-glucoside				
Quercetin 3-O-*β*-D-xylosyl-(1 ⟶ 2)-a-_L_-rhamnoside-4ʹ-O-a-_L_-rhamnoside				
Naringenin-6,8-di-C-hexoside	UHPLC-PDA-ESI-MS		Leaves	[2]
Quercetin-3-O-[(2-hexosyl)-6-rhamnosyl]-hexoside				
(Epi)catechin-di-C-hexoside				
Quercetin-3-O-robinoside				
Bayarin				
Quercetin-3-O-hexoside				
Quercetin-3-O-(2-pentosyl-rhamnoside)-4′-O-rhamnoside				
Quercetin-3-O-(4-O-*p*-coumaroyl)-2-rhamnosyl-[6-rhamnosyl]-galactoside				
Quercetin 3-O-rutinoside	UHPLC-PDA-ESI-MS, HPLC-PDA-MS, and NMR		Leaves	[2]
			Fruits	[173]
Quercetin 3-xylosyl-(1⟶2) rhamnoside-4′-rhamnoside	UV and NMR		Leaves	[175]
Quercitrin				
Gallic acid	HPLC		Leaves	[48]
	HPLC-DAD	5.09 ± 1.23	Pulp	[176]
		13.38 ± 1.66	Seed	
		3.00 ± 0.84	Almond	
Catechin	HPLC		Leaves	[48]
	HPLC-DAD	1.28 ± 1.66	Pulp	[176]
		10.98 ± 2.78	Seed	
Procyanidin B2	HPLC-DAD	63.22 ± 10.21	Pulp	[176]
		425.44 ± 11.35	Seed	
Chlorogenic acid	HPLC		Leaves	[48]
	HPLC-DAD	33.80 ± 2.66	Pulp	[176]
		15.0 ± 4.88	Seed	
		8.0 ± 1.77	Almond	
Cyanidin-3-galactosidase	HPLC-DAD	36.77 ± 4.12	Pulp	[176]
		131.78 ± 12.78	Seed	
		22.85 ± 2.60	Almond	
Caffeic acid	HPLC		Leaves	[48]
	HPLC-DAD	52.19 ± 17.02	Pulp	[176]
		576.33 ± 23.19	Seed	
		2.78 ± 0.92	Almond	
Anthocyanin	HPLC-DAD	1.27 ± 0.78	Pulp	[176]
		586.09 ± 34.77	Seed	
Epicatechin	HPLC		Leaves	[48]
	HPLC-DAD	11.33 ± 1.56	Pulp	[176]
		73.66 ± 12.66	Seed	
Cyanidin-3-rutinoside	HPLC-DAD	10.27 ± 0.80	Pulp	[176]
		43.88 ± 15.03	Seed	
*p*-Hydrobenzoic acid	HPLC-DAD	113.45 ± 11.30	Seed	[176]
Vanillic acid		13.79 ± 1.09		
Syringic acid	HPLC		Leaves	[48]
	HPLC-DAD	269.55 ± 22.89	Pulp	[176]
		210.04 ± 28.66	Seed	
Ferulic acid	HPLC-DAD	125.22 ± 11.67	Pulp	[176]
Sinapic acid	HPLC-DAD	171.88 ± 31.02	Pulp	[176]
		119.78 ± 10.55	Seed	
		185.67 ± 12.67	Almond	
Naringin	HPLC-DAD	1.23 ± 0.12	Pulp	[176]
		2.78 ± 0.78	Seed	
Rosmarinic acid	HPLC-DAD	222.18 ± 34.89	Pulp	[176]
		560.08 ± 35.28	Seed	
Hyperin	UV and NMR		Leaves	[175]
	HPLC-DAD	1.18 ± 0.19	Pulp	[176]
		2.11 ± 0.73	Seed	
Avicularoside	HPLC-DAD	19.23 ± 1.37	Seed	[176]
Resveratrol	HPLC-DAD	19.33 ± 4.86	Pulp	[176]
		22.60 ± 2.82	Seed	
Quercetin	HPLC/NMR and HRESIMS		Leaves	[48, 172]
	HPLC-DAD	25.08 ± 1.83	Pulp	[176]
		31.78 ± 6.88	Seed	
Volatile compounds				
*α*-Sitosterol	GC-MS	5.63	Leaves	[119]
5-Phenylundecane	GC-MS	10.88	Fruits	[25]
4-Phenylundecane		5.97		
3-Phenylundecane		5.41		
2-Phenylundecane		8.39		
6-Phenyldodecane		14.90		
4-Phenyldodecane		5.51		
3-Phenyldodecane		4.67		
2-Phenyldodecane		5.41		
6-Phenyltridecane		11.38		
2-Phenyltridecane		4.06		
Phytol	GC-MS	16.17	Leaves	[142]
Palmitic acid		24.66		
Oleic acid, omega 9		11.12		
Palmitoleic acid, methyl ester	GC-MS	4.84	Leaves	[142]
7-Octadecenoic acid, methyl ester	GC-MS, IR	19.63	Seeds	[177]
Methyl stearate		28.11		
Cis-11-eicosenoic acid, methyl ester		16.97		
Eicosanoic acid, methyl ester		4.97		
Docosanoic acid, methyl ester		10.76		
		8.60		
Geranyl acetone	GC and GC-MS	14.0	Leaves	[178]
Methyl hexadecanoate		10.0		
Methyl octadecenoate		9.9		
Farnesyl acetone		9.9		
Hexadecanol		9.7		
Ethyl octadecenoate		8.0		
Ethyl hexadecanoate		4.3		
Trihydroxy-octadecadienoic acid	UHPLC-PDA-ESI-MS		Leaves	[2]
Dihydroxydodecadienoic acid				
Trihydroxy-octadecenoic acid				
Amino-hexadecanediol				
Amino-methyl; heptadecantriol				
2-Amino-1,3-octadecanediol				
Octadecatetraenoic acid				
Triterpenic acid				
Oleanonic acid/bitulonic acid	UHPLC-PDA-ESI-MS		Leaves	[2]
Ceanothic acid				
Ceanothic acid isomer				
3-o-z-p-coumaroylalphitolic acid/3-o-z-p-coumaroylmaslinic acid				
Betulinic acid				
Alphitolic acid/maslinic acid				
Zizyberanalic acid/pomonic acid				
Other compounds				
Phloretin 3′/5′ di-c-galactoside	LC/ESI/MS		Fruits	[179]
Phloretin 3′-c-glucoside 5′-c-galactoside				
Cis-5-o-p-coumaroylquinic acid				
Diosmetin 3′-c-galactoside 7-o-rutinoside				
Diosmetin 3′-o-glucoside 7-o-rutinoside				
Trans-5-o-caffeoylquinic acid				
Trans-5-o-p-coumaroylquinic acid				

**Table 3:** Quantitative phytochemical data of *Z. spina*.

Plant part	Extract/fraction	Class of compounds	Quantity (%/mg/g/*μ*g/mL/mg/100 g)	References
Leaves	Methanol extract	Polyphenols (GAE)	74.6–58.7	[31]
		Flavonoids (QE)	64.2–47.3	
Leaves	Methanol extract	Quercetin	1.39–3.66	[183]
		Saponin	1.80–2.6	
Leaves	Methanol extract	Total flavonoids (RE)	710 ± 28.5	[142]
		Total phenolic content (GAE)	500 ± 10.5	
Roots	Aqueous extract	Total phenolic content (GAE)	59.89 ± 0.42	[41]
	Methanol extract	Total flavonoid content (QE)	9.01 ± 0.01	
	Ethanol extract	Catechin content (CE)	12.28 ± 1.07	
	Acetone extract	Total phenolic content (GAE)	59.98 ± 0.1	
		Total flavonoid content (QE)	9.45 ± 0.03	
		Catechin content (CE)	19.6 ± 0.19	
		Total phenolic content (GAE)	60.47 ± 0.04	
		Total flavonoid content (QE)	12.04 ± 0.04	
		Catechin content (CE)	23.98 ± 0.38	
		Total phenolic content (GAE)	60.26 ± 0.16	
		Total flavonoid content (QE)	11.64 ± 0.02	
		Catechin content (CE)	17.14 ± 2.01	
Aerial part	Methanol extract	Phenolics (GAE)	51.33 ± 0.4	[122]
		Flavonoids (QE)	14.78 ± 0.36	
		Tannins (CE)	21.6 ± 1.51	
Honey		Total phenolic (CE)	98.46 ± 2.24	[184]
Pulp	Aqueous extract	Total phenolic content (GAE)	30.97 ± 0.67	[176]
		Total flavonoid content (RE)	5.05 ± 1.09	
Seed		Total phenolic content (GAE)	74.46 ± 2.12	
		Total flavonoid content (RE)	19.84 ± 1.56	
Almond		Total phenolic content (GAE)	31.05 ± 1.30	
		Total flavonoid content (RE)	8.65 ± 0.78	
Honey		Total phenolic content (GAE)	20.15 ± 2.79–21.98 ± 0.70	[185]
Leaves	80% methanol extract	Total phenolic content (GAE)	87.3 ± 4.7	[186]
		Total flavonoid content (QE)	9.6 ± 1.1	

*Note*. GAE = gallic acid equivalent, QE = quercetin equivalent, RE = rutin equivalent, CE = catechin equivalent.

## Data Availability

The data are available within the manuscript.
